# Valvular dysfunction and left ventricular changes in Hodgkin's lymphoma survivors. A longitudinal study

**DOI:** 10.1038/sj.bjc.6605191

**Published:** 2009-07-21

**Authors:** T Wethal, M-B Lund, T Edvardsen, S D Fosså, A H Pripp, H Holte, J Kjekshus, A Fosså

**Affiliations:** 1 Department of Cardiology, Faculty Division Rikshospitalet, University of Oslo, Sognsvannsveien 20, 0027 Oslo, Norway; 2 Division of Cardiovascular and Respiratory Medicine and Surgery, Department of Respiratory Medicine, Rikshospitalet University Hospital and Faculty Division Rikshospitalet, University of Oslo, Sognsvannsveien 20, 0027 Oslo, Norway; 3 Department of Clinical Cancer Research, The Norwegian Radium Hospital and Faculty Division of the Norwegian Radium Hospital, University of Oslo, Montebello, N-0310 Oslo, Norway; 4 Biostatistics Unit, Department of Research Services, Rikshospitalet University Hospital, Sognsvannsveien 20, 0027 Oslo, Norway; 5 Cancer Clinic, The Norwegian Radium Hospital, Montebello, N-0310 Oslo, Norway

**Keywords:** lymphoma, cardiac valves, cardiomyopathy

## Abstract

**Purpose:**

Hodgkin's lymphoma survivors (HLSs) have an elevated risk for cardiovascular diseases that appear several years after radiotherapy. This study examined the time-dependent development and evolution of valvular and myocardial function related to treatment with mediastinal radiotherapy and anthracyclines in HLSs.

**Patients and methods:**

In 1993, echocardiography was performed in 116 HLSs median 10 years (range 6–13 years) after treatment with mediastinal radiotherapy. None of the 116 patients had valvular stenosis in 1993 whereas 36 (31%) had moderate valvular regurgitation. In 2005–2007, 51 of 57 invited patients were included in a second echocardiographic study – median 22 years (range 11–27 years) after treatment. Of these patients, 28 (55%) had also received anthracyclines. The patients were selected on the basis of the presence or absence of moderate valvular regurgitation in 1993.

**Results:**

The second echocardiographic study demonstrated that 10 out of 27 (37%) patients with only mild or no aortic or mitral regurgitation in 1993 had developed moderate regurgitation in either or both the aortic or mitral valve. Of the 24 patients with moderate (*n*=23) or severe (*n*=1) regurgitation in the aortic or mitral valve in 1993, 8 (33%) had progressed to severe regurgitation, developed moderate regurgitation in a previously normal or mild regurgitant valve or had received valvular replacement. In total, of all patients, 20 (39%) had developed mild to severe aortic stenosis and 3 patients had received valvular replacement. In a multiple linear regression the use of anthracyclines predicted left ventricular remodelling between ECHO 1993 and 2005 as demonstrated by increased left ventricular end systolic diameter (*β* =0.09 (95% CI 0.01–0.17), *P*=0.04) and reduced thickness of the left ventricular posterior wall (*β* =−0.18 (95% CI −0.33 to −0.03), *P*=0.02) and interventricular septum (*β* =−0.16 (95% CI −0.30 to −0.03), *P*=0.02).

**Conclusion:**

Given the progressive nature of valvular dysfunction and left ventricular remodelling 20–30 years after diagnosis, we recommend life-long cardiological follow-up of HLSs treated with mediastinal radiotherapy.

Hodgkin's lymphoma is one of the most common cancers in young adults; an annual incidence of 3 per 100 000 was recorded in the United States in 2004 (National Cancer Institute, 2008). Modern radiotherapy and chemotherapy used either alone or in combination have increased the 5-year overall survival rate to 85% (Boice, 2007). However, Hodgkin's lymphoma survivors (HLSs) have an elevated risk of developing long-term sequelae, including secondary malignancies and cardiovascular diseases (Hudson *et al*, 1998; Aleman *et al*, 2007; Heidenreich *et al*, 2007; Swerdlow *et al*, 2007). Several reports indicate that cardiovascular diseases appear more than 10 years after treatment, including coronary artery disease (CAD), congestive heart failure due to cardiomyopathy, valvular disease, and constrictive pericarditis (Lund *et al*, 1996; Hudson *et al*, 1998; Heidenreich *et al*, 2003, 2007; Hull *et al*, 2003; Adams *et al*, 2004; Aleman *et al*, 2007; Swerdlow *et al*, 2007). In addition, increased prevalences of calcification, stenosis in the great arteries, and stroke have been reported (King *et al*, 1999; Bowers *et al*, 2005; Apter *et al*, 2006; Patel *et al*, 2006). The main cause of cardiovascular disease in HLSs is thought to be radiotherapy. Furthermore, chemotherapy, especially anthracycline treatment, induces cardiomyopathy and aggravates cardiac disorders by various mechanisms (Singal and Iliskovic, 1998; Meinardi *et al*, 2000; Wallace, 2003; Wouters *et al*, 2005).

The incidence of valvular dysfunction has been reported to increase during the second decade after mediastinal radiotherapy for Hodgkin's lymphoma (Heidenreich *et al*, 2003; Hull *et al*, 2003; Adams *et al*, 2004). After 20 years, 6–15% of HLS have moderate or severe valvular regurgitation in the aortic or mitral valve, and a few present with aortic stenosis. In 1993, our group invited HLSs treated at the Norwegian Radium Hospital between 1980 and 1988 to perform echocardiography (ECHO 1993) if they fulfilled the following criteria: mediastinal radiotherapy (with or without chemotherapy), age <50 years at diagnosis, and relapse free survival for >5 years (Lund *et al*, 1996). Of the 116 patients participating (median observation time: 10 years), 36 (31%) demonstrated moderate regurgitation in 40 valves, primarily the aortic and mitral valves. Eighty individuals had no or mild regurgitation in 1993. No patients had developed valvular stenosis in 1993.

The cross-sectional nature of previously published studies makes it difficult to assess the time-dependent progression of valvular dysfunction in the individual patient. Therefore, during 2005 we performed a follow-up study with echocardiographic examinations in a subgroup of patients examined in 1993 by Lund *et al* (ECHO 2005) with the objective to assess valvular and ventricular changes between the examinations in 1993 and 2005. In 2005, our primary interest was to assess the post-1993 development of moderate regurgitations, conditions where regular echocardiographic follow-up is recommended according to guidelines (Bonow *et al*, 2006). Thus, all surviving patients with moderate and severe regurgitations in 1993 were recruited to participate in ECHO 2005. Mild regurgitation is frequently observed in the general population and not regarded as pathology. From the group of 80 patients without or with only mild valvular regurgitation we invited 25 age- and gender-matched survivors for comparison of changes.

## Patients and methods

### Patients

Of the 36 patients with moderate regurgitation in 1993, 4 had died (3 from cancer other than Hodgkin's lymphoma, 1 from CAD). A total of 57 patients were invited for ECHO 2005; all patients still alive with moderate regurgitation in 1993 (*n*=32) and 25 patients with no or mild regurgitation in 1993. Six patients declined. Of the 51 remaining participants, 28 had at least one valve with moderate or severe regurgitation at ECHO 1993. Four patients had either moderate pulmonal (*n*=2) or tricuspid (*n*=2) regurgitation but no or only mild aortic or mitral regurgitation. Thus, 24 patients had moderate mitral and/or aortic regurgitation whereas the remaining 27 patients demonstrated no (*n*=14) aortic or mitral regurgitation or only mild (*n*=13) aortic and/or mitral regurgitation. Before the reexamination at ECHO 2005 three patients had received mechanical valve implants (for aortic regurgitation, aortic stenosis, and mitral regurgitation, respectively), and for these patients the last preoperative echocardiography was included in the ECHO 2005 analysis as the second follow-up examination.

Disease and treatment characteristics were obtained from medical records, including type of radiotherapy (mantle field *vs* mediastinal field only) and details regarding the use of additional chemotherapy (Table 1). Radiotherapy was delivered in fractional doses of 1.8 or 2.0 Gy 5 days per week with a median dose of 40.0 Gy (range 24–44 Gy) to the mediastinum. No subcarinal blocks or cardiac shields were used. Anterior and posterior fields were weighted equally. Further treatment details are described elsewhere (Lund *et al*, 1996).

All patients completed a questionnaire regarding lifestyle parameters (smoking, physical activity) and underwent a clinical examination. Chronic medication, cardiovascular events, and cardiac risk factors were recorded. A cardiovascular event was defined as myocardial infarction, percutaneous coronary intervention or coronary artery bypass graft surgery, valvular surgery, stroke, or transitory ischaemic attack.

### Blood samples

Non-fasting blood was sampled between 0900 hours and 1200 hours. Plasma was stored at −70°C for later determination of CRP and pro-Brain Natriuretic Peptide (pro-BNP; Roche Diagnostics, Oslo, Norway), total cholesterol, low-density lipoprotein cholesterol (LDL), high-density lipoprotein cholesterol (HDL), and triglycerides.

### Echocardiography

We performed two-dimensional (2D) transthoracic echocardiography with a Vivid 7 scanner (GE Vingmed Ultrasound, Horten, Norway). Three consecutive heart cycles from the parasternal, long axis, short-axis, apical four chamber, and subcostal views were obtained. The digital loops were stored and analysed by EchoPac software (GE Vingmed Ultrasound). In M-mode, we recorded measurements of left atrial (LA) diameter, left ventricular end-diastolic and end-systolic diameters (LVEDD and LVESD, respectively), left ventricular posterior wall (LVPW), interventricular septum thickness (IVSd), and left ventricular shortening fraction (LV-SF). Cardiac index (CI) was calculated from cardiac stroke volume, body height, and body weight. Left ventricular ejection fraction (LVEF) was assessed by the modified Simpson's rule. LVEF and left ventricular end diastolic volume were measured only in ECHO 2005. 2D and pulsed Doppler echocardiography were used to estimate valvular regurgitation and stenosis (mild, moderate, or severe) according to published recommendations (Zoghbi *et al*, 2003; Bonow *et al*, 2006). Left ventricular remodelling was defined as a thinning of ventricular walls and dilatation of the ventricle but without any impairment in the systolic function between ECHO 1993 to 2005. All examinations were carried out according to a standardised protocol by physicians and sonographers at the Department of Cardiology, and all the data were interpreted by a single experienced cardiologist, blinded for patient history (including the results from ECHO 1993) and treatment.

### Statistics

Data were expressed as means/medians with standard deviations (s.d.)/range or as percentages. The Student's *t*-test was used to compare normally distributed continuous data. The Mann–Whitney test was used for highly skewed continuous data. The *χ*^2^-test was performed to compare categorical data between groups. Differences in left ventricular function between ECHO 1993 and ECHO 2005 were tested with the paired-samples *t*-test. Furthermore, for each patient, the changes (expressed as percentage increase or decrease) in left ventricular parameters from ECHO 1993 to ECHO 2005 were calculated. The patients were then grouped according to the previous use of anthracyclines, the presence of aortic stenosis, cardiovascular disease, and gender. Intergroup differences in echocardiographic parameters were analysed with the independent-samples *t*-test. Pearson correlation analysis was used to investigate the relationships between age, pro-BNP, and CRP and echocardiographic variables. Multiple linear regression analyses were performed with significant univariates (*P*<0.10) as independent variables and with the percent changes in each of the six echocardiographic variables as dependent variables. Binary logistic regression was performed to investigate whether any of the treatment-related or clinical parameters were associated with aortic stenosis or new onset valvular regurgitation. A two-tailed *P* value ⩽0.05 was considered statistically significant. All statistical analyses were performed with SPSS 14.0 (SPSS Inc., Chicago, IL, USA). The study was approved by the institution's protocol review committee. All the participants gave their informed consent.

## Results

Hodgkin's lymphoma had been diagnosed in stages I and II in 40 (78%) patients and in stages III and IV in 11 (22%) patients (Table 1). The median age at diagnosis was 26 years (range 14–42 years), and 67% of the participants were women. Mantle or mediastinal fields had been given to 43 and 8 of 51 patients, respectively. The median dose to the mediastinum was 40.0 Gy (range 27–44 Gy). A total of 36 patients received additional chemotherapy, either as part of their primary treatment together with radiotherapy or, in 6 patients (12%), as treatment for relapse. Anthracyclines were given to 28 (55%) patients with a total median adriamycin dose of 320 mg (range 110–480 mg) in 27 and 720 mg of epirubicin in 1 patient. The median time between diagnosis and the second echocardiography was 22 years (range 11–27 years). At ECHO 2005, 15 patients reported a preceding cardiovascular event.

### Valvular dysfunction

#### Valvular regurgitation

Of 14 patients with no evidence of aortic or mitral regurgitation in 1993, only 1 remained unchanged at ECHO 2005 (Table 2). Nine patients had developed only mild whereas three developed moderate regurgitation in the aortic, mitral valve or both, and one patient had an aortic valve implantation due to aortic stenosis.

There were 13 patients with only mild regurgitation in either the mitral or aortic valve in 1993. Only two patients remained unchanged with respect to valvular regurgitation, but one developed aortic stenosis. Four patients developed mild regurgitation in a previously unaffected valve and seven patients had progressed to moderate mitral and/or aortic regurgitation at ECHO 2005.

At ECHO 2005, 10 of the 27 patients (37%) without or with only mild valvular regurgitation progressed to moderate regurgitation in the aortic and/or mitral valve. In addition, 13 had new aortic stenosis.

Twenty-three patients had moderate regurgitation in either or both the aortic or mitral valve in 1993. Status remained unchanged in 10 patients at ECHO 2005. Two patients had progressed to a new moderate regurgitation in either the aortic or mitral valve. Four patients went from moderate to severe regurgitation and one had an aortic valve implantation due to regurgitation. One patient had severe mitral regurgitation at ECHO 1993, which was treated with a mechanical valve during follow-up. Thus, of the 24 patients with a moderate or severe regurgitation in the aortic or mitral valve in 1993, 8 (33%) had progressed to severe regurgitation, to moderate regurgitation in another previous normal valve, or had performed valvular surgery.

#### Aortic stenosis

**Figure 1:**
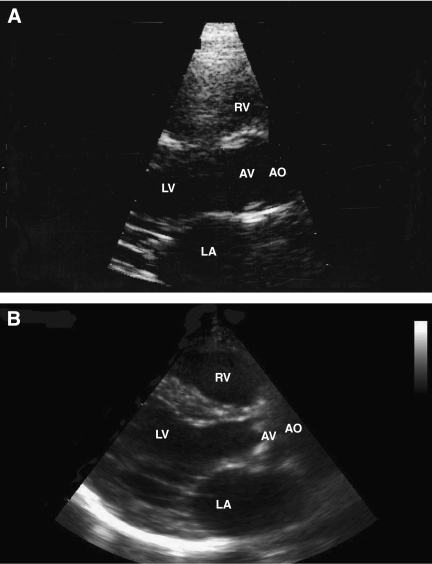
(**A**) In 1993 (7.5 years postradiotherapy), the aortic valve was without pathology and opened completely . (**B**) In 2007 (21.5 years postradiotherapy), echocardiography showed a thickened aortic valve, which hardly opened during ventricular systole in a patient with severe aortic stenosis. Abbreviations: AO, aorta; AV, aortic valve; LV, left ventricle; LA, left atrium; RV, right ventricle.

No aortic stenosis was demonstrated in 1993. In contrast, at ECHO 2005, 20 out of 51 (39%) patients had developed aortic stenosis (mild (13), moderate (3), and severe (4)) with an estimated mean aortic valve area of median 1.6 cm^2^ (range 0.6–3.2 cm^2^) and a mean valvular gradient of 11 mmHg (range 5–45 mmHg) (Figure 1).

Binary logistic regression with the presence of aortic stenosis as the dependent variable failed to show any relationship with chemotherapy, the presence or absence of valvular regurgitation in 1993 or any of the clinical parameters given in Table 1. New-onset valvular regurgitation did not demonstrate any relation to clinical or treatment related parameters.

#### Appearance of the valves

The appearance of both the aortic and mitral valve was dominated by different degrees of degeneration including leaflet thickening, reduced leaflet movements, and calcification. Calcification was observed among those with moderate/severe stenosis or regurgitation. The mean ejection fraction was 54% (s.d. 7%) and mean left ventricular end diastolic volume was 97 ml (s.d. 31 ml) indicating that mitral regurgitation was due to organic involvement of the valve. Only two patients had EF below 45% and these patients had moderate aortic regurgitation and mild mitral regurgitation, respectively.

### Left ventricular remodelling

Compared to ECHO 1993, the second echocardiography demonstrated an increased LVESD (*P*=0.02) and a reduced LV-SF (*P*=0.003; Table 3). Additionally, LA diameter was increased for all participants in ECHO 2005 (*P*<0.001).

Multiple linear regression analyses were performed with previous use of anthracyclines, age at ECHO 2005, cardiovascular disease, and the presence of aortic stenosis as independent variables. The use of anthracyclines was associated with increased LVESD and impaired IVSd and LVPW indicating left ventricular remodelling (Table 4). Additionally, increasing age at ECHO 2005 was associated with an independent effect on IVSd and CI. Demonstration of aortic stenosis was associated with increased LVEDD and LVESD.

### Biochemical markers

CRP and pro-BNP was higher among patients with moderate or severe aortic or mitral regurgitation in ECHO 2005 compared to patients without valvular regurgitation (CRP: median 2.5 mg l^−1^ (range 0.2–43 mg l^−1^) and 1.1 mg l^−1^ (0.6–8.8 mg l^−1^), *P*=0.015; pro-BNP: median 24 pmol l^−1^ (range 4–201 pmol l^−1^) and 10 pmol l^−1^ (range 4–53 pmol l^−1^), *P*=0.006). BNP correlated positively with LVEDD (*r*=0.36, *P*=0.01), LVESD (*r*=0.38, *P*=0.007) and negatively with EF (*r*=−0.29, *P*=0.045).

## Discussion

This study clearly demonstrates a frequent and progressive valvular deterioration during the second decade after mediastinal radiotherapy for Hodgkin's lymphoma. Although the population studied is biased towards patients with moderate valvular regurgitation already present 10 years after treatment at ECHO 1993, 10 of 27 (37%) patients with no or mild aortic or mitral regurgitation had in ECHO 2005 developed moderate aortic and/or mitral regurgitation. Furthermore, after a median observation time of 22 years, aortic stenosis was observed in 39%, developed after 1993 independently of the presence of regurgitation at ECHO 1993. Even if the use of anthracyclines was associated with left ventricular remodelling in the second decade after treatment this study did not show any interaction between anthracyclines and the development of valvular dysfunction.

### Valvular dysfunction

Mediastinal radiotherapy is a known risk factor for the development of cardiovascular diseases affecting the coronary arteries, pericardium, myocardium, conduction system, and myocardial valves (Lund *et al*, 1996; Hudson *et al*, 1998; Heidenreich *et al*, 2003, 2007; Hull *et al*, 2003; Adams *et al*, 2004; Aleman *et al*, 2007; Swerdlow *et al*, 2007). One precipitating factor is believed to be endothelial dysfunction, caused by an active cellular process involving chronic inflammation resulting in reduced flow-mediated vasodilatation in arteries within the radiation field (Sugihara *et al*, 1999; Beckman *et al*, 2001). This dysfunction most likely comprises reduced tissue endothelial nitric oxide synthase, stimulation of growth factors, and fibrosis. Radiotherapy may also directly damage the valves and cause fibrotic thickening, retraction, and calcifications (Basavaraju and Easterly, 2002). More recently, decreased levels of endothelial progenitor cells has been suggested to reduce endothelial regenerative capacity and to contribute to the progression of degenerative aortic stenosis (Matsumoto *et al*, 2009). Though valves are normally avascular, cellular injury may cause fibrosis and stimulate secondary angiogenesis and calcification. Our results suggest that valve retraction is the predominant early change that causes valvular regurgitation, and it takes as long as 20 years to develop thickened, calcified valves that may finally result in stenosis. Higher pressure on the left side of the heart likely explains the observation that the aortic and mitral valves were affected more often than the tricuspid or pulmonary valves, and that stenosis is primarily observed in the aortic valve (Heidenreich *et al*, 2003; Hull *et al*, 2003; Adams *et al*, 2004). Previous reports have demonstrated that valvular calcification is a dominant finding after mediastinal radiotherapy for Hodgkin's lymphoma (Adabag *et al*, 2004; Apter *et al*, 2006). This study suggests that this occurs later in the development of valvular pathology. Valvular calcification was seen among those with moderate/severe valvular regurgitation/stenosis whereas leaflet thickening and reduced movements without calcification were seen among those with no or only mild valvular stenosis/regurgitation. There was no evidence in this study of incipient stenosis of the mitral valve.

CRP is a marker of future CAD and a level above 2.0 mg l^−1^ has been shown to be associated with an increased risk of CAD (Ridker *et al*, 2005). CRP was generally elevated in our group of HLSs, especially in those with moderate and severe regurgitation reflecting that a lasting inflammatory condition is initiated after radiotherapy.

Pro-BNP as a marker of heart failure was elevated among patients with moderate and severe valvular regurgitation, and correlated positively with left ventricular end-systolic andend-diastolic diameter as well as with decreased EF. None of the patients had symptomatic heart failure, but valvular regurgitation and signs of left ventricular remodelling sufficient to cause elevated pro-BNP was a delayed consequence of treatment for Hodgkin's lymphoma.

The actual risk of valvular dysfunction in patients treated for Hodgkin's lymphoma is under debate. Aleman *et al* (2007) observed a hazard ratio of 7.0 and a cumulative incidence of approximately 10% for having clinically diagnosed valvular disorder after a median observation time of 13 years in HLSs treated with mediastinal radiotherapy. Hull *et al* (2003) reported that 6% of 415 HLSs had clinically important valvular disorder 20 years after radiotherapy. Heidenreich *et al*, (2003) performed a cross-sectional follow-up investigation in 73 patients 20 years after radiotherapy with 12 (16%) cases of aortic stenosis, 11 (15%) cases of moderate or severe aortic regurgitation, and 3 (4%) cases of mitral regurgitation. This study demonstrates higher rates of valvular regurgitation and aortic stenosis. These differences may be due to study design (eg, identification of symptomatic disease or screening methods), treatment differences (eg, field weighing, shielding, use of anthracyclines), variable classification of valvular dysfunction, varying sample sizes, and recruitment bias as well as duration of follow-up. In particular, 24 patients in this study were selected on the basis of their known moderate aortic and/or mitral valvular regurgitation 10 years after treatment. However, even among the 27 patients with no or mild aortic or mitral regurgitation at 10 years of follow-up, we found comparable and elevated rates of moderate valvular regurgitation (39%) which is higher than expected from previous studies. Lund *et al* (1996) found moderate valvular regurgitation to be more frequent in women than in men. The high prevalence of valvular dysfunction in this study might, at least in part, be due to the high percentage of women (67%). This study demonstrated no additional effect of anthracyclines on the development of valvular dysfunction although this has been reported by another study (Aleman *et al*, 2007). Our study indicates that deterioration of valvular regurgitation and the development of aortic stenosis are slow dynamic processes that may appear more than 10 years after the diagnosis of Hodgkin's lymphoma. Importantly we could show that all aortic stenosis developed after 1993 during the second and third posttreatment decade.

The fact that patients had been informed about their individual findings in ECHO 1993 may theoretically have changed the disease course in our HLSs studied in ECHO 2005. The evolution of valvular disease in patients with moderate regurgitation in 1993 may have been modified by treatment advice as a consequence of the findings. We have no information about eventual post-1993 lifestyle adjustments and medical treatment in our patients. Nevertheless, the majority of our patients were not followed systematically with echocardiography as isolated moderate regurgitation or valvular stenosis in the absence of heart failure was not treated medically. None, except the three patients with valvular replacement, had symptomatic heart failure at the examination in ECHO2005. Conversely, patients with no or mild regurgitation in 1993 may have paid less attention to lifestyle and cardiac symptoms until ECHO 2005, provoking an acceleration of the disease course. This suggestion is not supported by our data: patients without or mild compared to moderate regurgitation showed similar characteristics regarding comorbidities, cardiac risk factors, and intervention for hypertension and hypercholesterolaemia. We therefore believe that our longitudinal findings, to a large extent, mirror the ‘natural course’ of valvular function after mediastinal radiotherapy for Hodgkin's lymphoma.

### Left ventricular function

Anthracyclines are associated with dose-dependent cardiotoxicity, characterised by dilated cardiomyopathy and incipient heart failure (Singal and Iliskovic, 1998; Meinardi *et al*, 2000; Belham *et al*, 2007). The mechanisms underlying anthracycline-associated myocardial damage are undefined, but a potential mechanism may include enhanced apoptosis of cardiomyocytes (Meinardi *et al*, 2000; Wallace, 2003; Wouters *et al*, 2005). Our results consistently demonstrated that treatment with low to intermediate doses of anthracyclines were associated with thinning of ventricular walls combined with an increase in ventricular dilatation compared to radiotherapy alone or radiotherapy combined with chemotherapy without anthracyclines. This is of relevance for the treatment principles of stages I and II Hodgkin's lymphoma even today, as short-term anthracycline-based chemotherapy followed by involved field radiotherapy (frequently encompassing parts of the mediastinum) is still considered standard treatment for early-stage disease in most Western countries (Ferme *et al*, 2007). To our knowledge, this is one of the first studies to document a progressive unfavourable effect of anthracyclines on cardiac function two decades after the treatment of adult onset cancer. Studies are underway which will evaluate the time-dependent changes in cardiac function and examine the effect of an ACE-inhibitor on myocardial performance, mainly in children with different types of leukaemia and lymphomas (Singh *et al*, 1999; Silber *et al*, 2004; Lipshultz *et al*, 2005).

### Limitations

This study is limited by the small sample size and lack of a control group of healthy individuals. This makes it difficult to estimate the influence of age and other confounding factors and to relate our findings to the general population. The Framingham population between the ages of 40 and 60 years (Singh *et al*, 1999) displayed a 1% prevalence of moderate or severe mitral regurgitation in both men and women, and a 0.4 and 0.1% prevalence of moderate or severe aortic regurgitation in men and women, respectively. The Framingham population does not directly correspond to the Norwegian population. Nevertheless, our data, though biased towards patients with valvular regurgitation 10 years after the diagnosis of Hodgkin's lymphoma, suggest an elevated risk of premature valvular dysfunction among HLSs, not at least if compared to Lund *et al*'s control group from 1993 where none of 40 age- and gender-matched controls displayed moderate valvular regurgitation. The strength of our study was the longitudinal follow-up design that enabled documentation of the slow progressive nature of valvular dysfunction in individual patients independent of valvular involvement at the first examination.

### Conclusions

This study indicates that HLS treated with mediastinal radiotherapy are at a high risk of developing progressive valvular deterioration and subsequent aortic stenosis during the second or third decade after treatment. Additional treatment with anthracyclines is associated with left ventricular remodelling. These findings suggest that a strategy for cardiological follow-up should be implemented for these patients considering the slowly evolving cardiovascular side effects of radiotherapy and chemotherapy in HLSs.

## Conflict of interest

The authors declare no conflict of interest.

## Figures and Tables

**Table 1:** Demographic and clinical characteristics of the study group

**Number of patients (*n*=51)**	
*Age (years)*	
At cancer diagnosis	26 (14–42)
At current examination	50 (35–63)
Observation time	22 (11–27)
Females; *n* (%)	34 (67)
	
*Cancer stage;* n (%)	
Stages I and II	40 (78)
Stages III and IV	11 (22)
Relapses; *n* (%)	6 (12)
	
*Radiotherapy*	
Radiation dose (Gy)	40.0 (27.0–44.0)
Mantle field; *n* (%)	43 (84)
Mediastinal field only; *n* (%)	8 (16)
	
*Chemotherapy*	
Chemotherapy; *n* (%)^a^	36 (71)
Received anthracyclines; *n* (%)	28 (55)
Dose of adriamycin (mg)^a^	320 (110–480)
ChlVPP/ABOD; *n* (%)	23 (45)
ChlVPP only; *n* (%)	5 (10)
ABOD only; *n* (%)	4 (8)
ChlVPP/EBVP; *n* (%)^b^	1 (2)
Other regimes; *n* (%)	3 (6)
Current smoker; *n* (%)	14 (27)
	
*Self-reported comorbidities;* n (%)	
History of hypothyroidism	34 (67)
History of hypercholesterolaemia	10 (20)
Diabetes	1 (4)
Cardiovascular disease	15 (29)
Coronary disease	9 (18)
Stroke or TIA	3 (6)
Valvular surgery	3 (6)
	
*Clinical findings*	
Systolic blood pressure (mmHg)	123 (85–156)
Diastolic blood pressure (mmHg)	72 (52–95)
Hypertension; *n* (%)^c^	13 (25)
Body mass index (kg/m^2^)	24.0 (17.4–34.2)
	
*Blood samples*	
Cholesterol (mmol l^−1^)	5.3 (3.4–7.0)
HDL cholesterol (mmol l^−1^)	1.3 (0.8–2.3)
LDL-cholesterol (mmol l^−1^)	3.6 (1.8–4.7)
Triglycerides (mmol l^−1^)	1.1 (0.6–3.7)
CRP (mg l^−1^)	2.35 (0.20–43)
proBNP (pmol l^−1^)	21.0 (3.7–201)

Medians and ranges are given for all continuous parameters.

ChlVPP=chlorambucil, vinblastin, procarbazine, prednisolon; ABOD=adriamycin, bleomycin, vincristin, dacarbazine; EBVP=epirubicin, bleomycin, vinblastin, prednisolon.

a Including chemotherapy for relapses.

b Epirubicin was given to one patient at a total dose of 720 mg.

c Hypertension: systolic blood pressure ⩾140 mmHg or a diastolic blood pressure ⩾90 mmHg or the use of antihypertensive medication in order to lower blood pressure.

**Table 2:** The development of aortic regurgitation, mitral regurgitation, and aortic stenosis from 1993 to 2005–07

		**ECHO 2005**		
**ECHO 1993 aortic or mitral regurgitation**		**New or persistent aortic or mitral regurgitation**		**New aortic stenosis**
No	14^a^	Status unchanged	1	
		Mild only (new)	9^a^	4
		Moderate (new)	3	1
		Implanted valve	1^a^	1
				
Mild	13^b^	Status unchanged	2	1
		Mild only (new)	4	1
		Moderate (new)	7^b^	5
Moderate	23	Status unchanged	10	3
		*Moderate*		
		New	2	2
		Persistent+new mild	6	2
		Severe (new)	4	
		Implanted valve	1	
Severe	1	Implanted valve	1	
Total	51		51	20

a Also includes two patients with moderate pulmonary regurgitation and one with moderate tricuspid regurgitation.

b Also includes one patient with moderate tricuspid regurgitation.

**Table 3:** Echocardiographic examinations

**Number of patients (*n*=51)**	**ECHO 1993**	**ECHO 2005**	***P* value**
LA (cm)	3.1 (0.6)	3.4 (0.6)	<0.001
LVEDD (cm)	4.8 (0.5)	4.9 (0.5)	0.70
LVESD (cm)	3.2 (0.4)	3.4 (0.6)	0.02
IVSd (cm)	0.9 (0.2)	0.9 (0.2)	0.80
LVPW (cm)	0.8 (0.2)	0.8 (0.2)	0.78
LV-SF (%)	34.2 (4.7)	31.2 (6.3)	0.003
CI, (l min^−1^ m^−2^)	2.9 (0.5)	3.0 (0.7)	0.57
EF (%)		54 (7)	
LVEDV (ml)		97 (31)	

Means (standard deviations) are given for all continuous parameters.

LA=left atrium diameter; LVEDD=left ventricular end diastolic diameter; LVESD=left ventricular end systolic diameter; IVSd=interventricular septum thickness; LVPW=left ventricular posterior wall; LV-FS=left ventricular shortening fraction; CI=cardiac index; EF=ejection fraction; LVEDV=left ventricular end diastolic volume.

**Table 4:** Multiple linear regression analysis of factors involved in changes of left ventricular function from ECHO 1993 to ECHO 2005

	**Treatment with anthracyclines (yes/no)**		**Age at ECHO 2005**		**Cardiovascular disease (yes/no)**		**Aortic stenosis (yes/no)**	
**Echocardiographic parameters**	***β* (95 % CI)**	***P* value**	***β* (95 % CI)**	***P* value**	***β* (95 % CI)**	***P* value**	***β* (95 % CI)**	***P* value**
LVEDD	0.051 (−0.001 to 0.102)	0.053	0.003 (−0.001 to 0.007)	0.15	0.018 (−0.043 to 0.078)	0.56	0.060 (0.009 to 0.111)	0.021
LVESD	0.089 (0.006 to 0.172)	0.036	0.005 (−0.001 to 0.012)	0.091	0.087 (−0.011 to 0.185)	0.082	0.079 (−0.003 to 0.161)	0.058
IVSd	−0.161 (−0.297 to −0.025)	0.021	−0.010 (−0.021 to 0.000)	0.046	0.040 (−0.121 to 0.201)	0.62	0.004 (−0.130 to 0.139)	0.95
LVPW	−0.178 (−0.325 to −0.031)	0.019	−0.005 (−0.016 to 0.006)	0.35	−0.010 (−0.185 to 0.164)	0.91	0.020 (−0.125 to 0.166)	0.78
LV-SF	−0.058 (−0.176 to 0.060)	0.33	−0.004 (−0.013 to 0.005)	0.38	−0.168 (−0.308 to −0.028)	0.020	−0.008 (−0.125 to 0.109)	0.89
CI	0.018 (−0.156 to 0.191)	0.84	−0.014 (−0.028 to −0.001)	0.035	−0.154 (−0.359 to 0.051)	0.14	0.056 (−0.116 to 0.227)	0.52

LVEDD=left ventricular end diastolic diameter; LVESD=left ventricular end systolic diameter; IVSd=interventricular septum thickness; LVPW=left ventricular posterior wall; CI=cardiac index; LV-FS=left ventricular shortening fraction.
